# Cannabis sativa phytoligands as potential EGFR inhibitors for lung cancer treatment

**DOI:** 10.21203/rs.3.rs-11024152/v1

**Published:** 2026-10-13

**Authors:** Afolabi Owoloye, Precious A. Akinnusi, Samuel O. Olubode, Evans A. Adu, Natalia Shakela, David O. Oyerinde, Olamide L. Onawola, Kolapo M. Oyebola

## Abstract

Lung cancer remains a major global health burden, and epidermal growth factor receptor (EGFR) overexpression promotes tumour-cell proliferation, making EGFR an important therapeutic target. This study used an *in-silico* approach to evaluate *Cannabis sativa* compounds as inhibitors of the EGFR kinase domain. Among the compounds investigated, Luteolin-7 *-O-* glucoside, grossamide, and quercetin were the leading ATP-competitive inhibitors, with binding affinities of -10.878, -10.091, and − 10.000 kcal/mol, respectively. A four-feature pharmacophore model was generated from the lead compounds, which also showed favourable predicted pharmacokinetic profiles. During 100-ns molecular dynamics simulations (MDS), the EGFR-quercetin complex exhibited the lowest root mean square deviation of 0.9–1.5 Å, while fluctuation profiles revealed a relatively stable ligand-bound complex compared with the apo-EGFR. The MDS trajectory Molecular Mechanics/Generalized Born Surface Area analysis yielded binding free energies of -75.59 kcal/mol for grossamide, -56.90 kcal/mol for isovitexin, -49.11 kcal/mol for luteolin-7 *-O-* glucoside, and − 43.27 kcal/mol for quercetin, compared with − 47.41 kcal/mol for gefitinib. Electronic profiling revealed favourable orbital overlap and softness for quercetin and flavone glycosides within EGFR, while isovitexin showed superior kinetic stability and balanced solvation, supporting strong binding and active-site entry. These findings identify luteolin-7 *-O-* glucoside, grossamide, and quercetin as promising EGFR inhibitors and provide a baseline hypothesis for experimental validation.

